# Methyl 2-(4,6-dichloro-1,3,5-triazin-2-yl­amino)acetate

**DOI:** 10.1107/S1600536809028670

**Published:** 2009-07-25

**Authors:** Sérgio M. F. Vilela, Filipe A. Almeida Paz, João P. C. Tomé, Verónica de Zea Bermudez, José A. S. Cavaleiro, João Rocha

**Affiliations:** a Department of Chemistry, University of Aveiro, CICECO, 3810-193 Aveiro, Portugal; b Department of Chemistry, University of Aveiro, QOPNA, 3810-193 Aveiro, Portugal; c Department of Chemistry, CQ-VR, University of Trás-os-Montes e Alto Douro, 5001-801 Vila Real, Portugal

## Abstract

The title compound, C_6_H_6_Cl_2_N_4_O_2_, was prepared by the nucleophilic substitution of 2,4,6-trichloro-1,3,5-triazine by glycine methyl ester hydro­chloride, and was isolated from the reaction by using flash chromatography. The crystal structure at 150 K reveals the presence two crystallographically independent mol­ecules in the asymmetric unit which differ in the orientation of the pendant methoxy­carbonyl group. Each mol­ecular unit is engaged in strong and highly directional N—H⋯N hydrogen-bonding inter­actions with a symmetry-related mol­ecule, forming supra­molecular dimers which act as the synthons in the crystal packing.

## Related literature

For background to nucleophilic reactions based on 1,3,5-triazine derivatives, see: Blotny (2006 ▶); Giacomelli *et al.* (2004 ▶). For coordination polymers based 1,3,5-triazine derivatives, see: Wang, Xing *et al.* (2007 ▶); Wang, Bai, Xing *et al.* (2007 ▶); Wang, Bai, Li *et al.* (2007 ▶). For general background studies on crystal-engineering approaches from our research group, see: Vilela *et al.* (2009 ▶); Shi *et al.* (2008 ▶); Paz & Klinowski (2003 ▶, 2007 ▶); Paz *et al.* (2002 ▶, 2005 ▶). For a description of the graph-set notation for hydrogen-bonded aggregates, see: Bernstein *et al.* (1995 ▶). For a description of the Cambridge Structural Database and the Mercury software package, see: Allen (2002 ▶); Macrae *et al.* (2008 ▶).
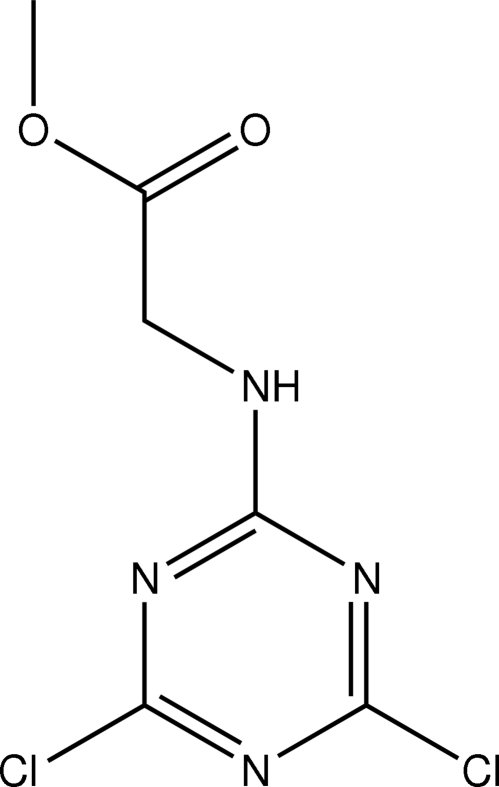


## Experimental

### 

#### Crystal data

C_6_H_6_Cl_2_N_4_O_2_
*M* *_r_* = 237.05
Triclinic, 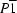

*a* = 7.3543 (4) Å
*b* = 9.7523 (5) Å
*c* = 13.4133 (7) Å
α = 97.714 (3)°
β = 92.714 (3)°
γ = 90.225 (3)°
*V* = 952.19 (9) Å^3^
*Z* = 4
Mo *K*α radiation
μ = 0.66 mm^−1^
*T* = 150 K
0.18 × 0.16 × 0.04 mm

#### Data collection

Bruker X8 Kappa CCD APEXII diffractometer
Absorption correction: multi-scan (*SADABS*; Sheldrick, 1997 ▶) *T*_min_ = 0.890, *T*_max_ = 0.974
23605 measured reflections
5043 independent reflections
3753 reflections with *I* > 2σ(*I*)
*R*_int_ = 0.047

#### Refinement

*R*[*F* ^2^ > 2σ(*F* ^2^)] = 0.059
*wR*(*F* ^2^) = 0.165
*S* = 1.04
5043 reflections
261 parameters
2 restraints
H atoms treated by a mixture of independent and constrained refinement
Δρ_max_ = 1.78 e Å^−3^
Δρ_min_ = −0.42 e Å^−3^

### 

Data collection: *APEX2* (Bruker, 2006 ▶); cell refinement: *SAINT-Plus* (Bruker, 2005 ▶); data reduction: *SAINT-Plus*; program(s) used to solve structure: *SHELXTL* (Sheldrick, 2008 ▶); program(s) used to refine structure: *SHELXTL*; molecular graphics: *DIAMOND* (Brandenburg, 2009 ▶); software used to prepare material for publication: *SHELXTL*.

## Supplementary Material

Crystal structure: contains datablocks global, I. DOI: 10.1107/S1600536809028670/tk2507sup1.cif

Structure factors: contains datablocks I. DOI: 10.1107/S1600536809028670/tk2507Isup2.hkl

Additional supplementary materials:  crystallographic information; 3D view; checkCIF report

**Fig. 5.**
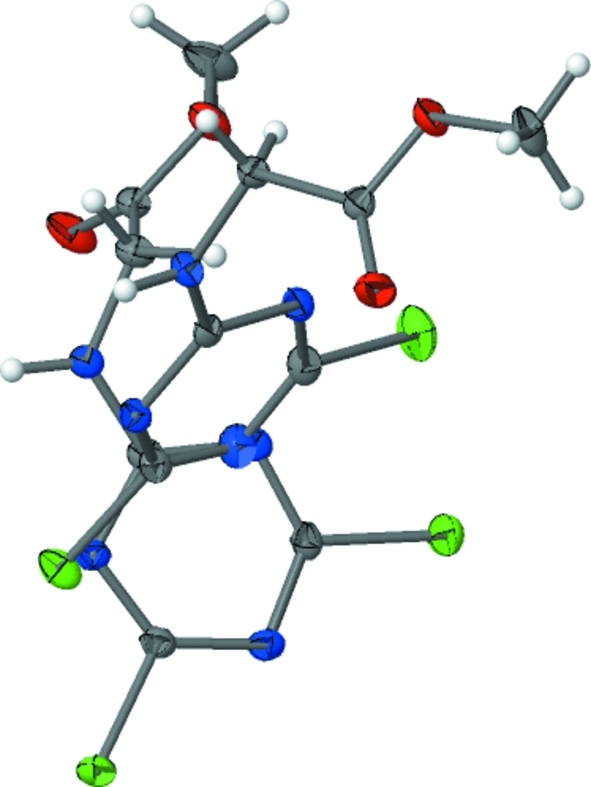
Asymmetric unit of the title compound depicting the two crystallographically independent molecular units. Non-hydrogen atoms are represented as thermal ellipsoids drawn at the 50% probability level.

Enhanced figure: interactive version of Fig. 5

**Fig. 6.**
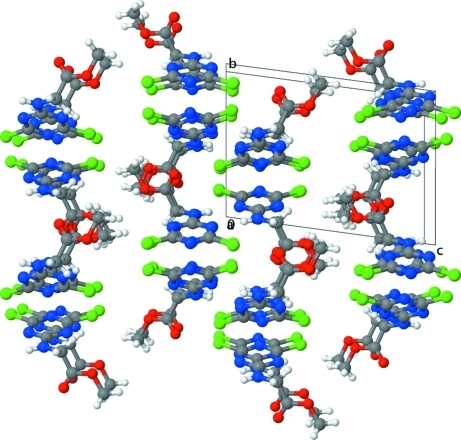
Crystal packing of the title compound viewed in perspective along the [100] direction of the unit cell.

Enhanced figure: interactive version of Fig. 6

## Figures and Tables

**Table 1:** Hydrogen-bond geometry (Å, °)

*D*—H⋯*A*	*D*—H	H⋯*A*	*D*⋯*A*	*D*—H⋯*A*
N4—H4⋯N2^i^	0.945 (10)	2.092 (12)	3.028 (3)	171 (3)
N8—H8⋯N6^ii^	0.943 (10)	2.083 (11)	3.022 (3)	173 (3)

Symmetry codes: (i) 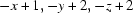; (ii) 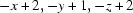.

**Fig. 1.**
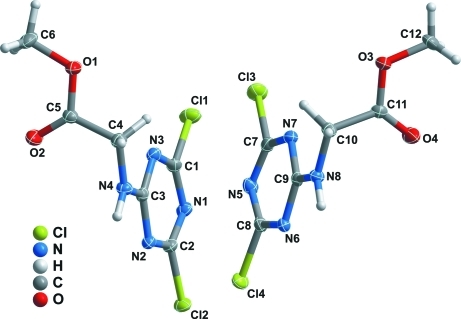
Molecular structures of the two independent molecules in (I). Non-hydrogen atoms are represented as thermal displacement ellipsoids drawn at the 50% probability level and hydrogen atoms as small spheres with arbitrary radii. The atomic labeling is provided for all non-hydrogen atoms.

**Fig. 2.**
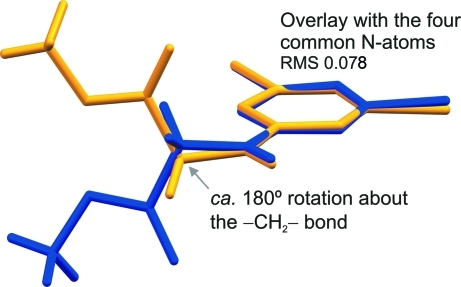
Structure overlay of the two crystallographically independent molecular units comprising the asymmetric unit in (I): while the —NH group remains almost co-planar with the aromatic ring, the two methoxycarbonyl groups are mutually rotated by ca 180° around the —CH2— bond.

**Fig. 3.**
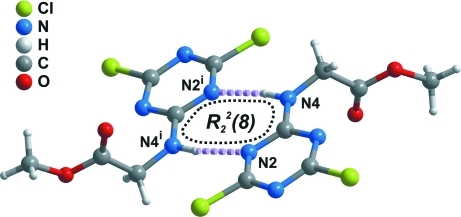
Strong N—H···N hydrogen bonding interactions connecting adjacent molecular units via a R22(8) synthon. For details on the hydrogen bonding geometry see Table 1.

**Fig. 4.**
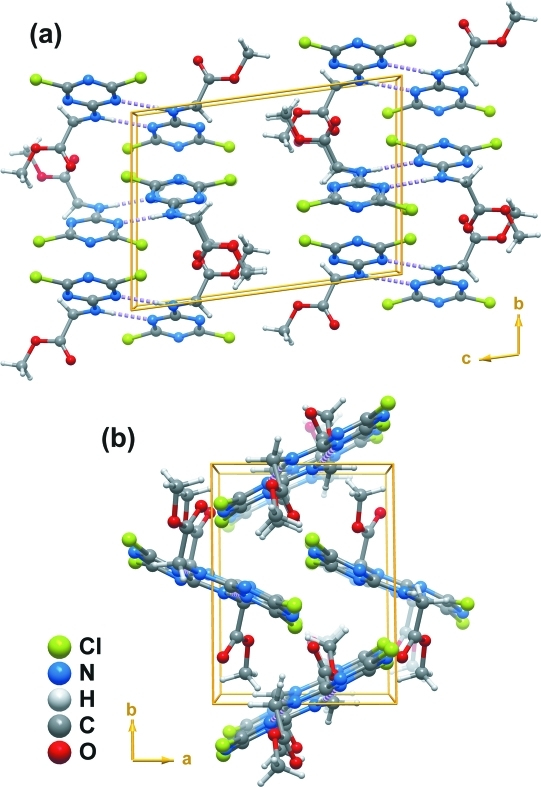
Crystal packing of (I) viewed in perspective along the (a) [100] and (b) [001] directions of the unit cell. N—H···N hydrogen bonds are represented as violet dashed lines.

## supplementary crystallographic information

### Comment

Worldwide research on 1,3,5-triazine derivatives has increased quite considerably in recent years driven by the versatility of this molecule which allows the nucleophilic substitution of the chloride atoms by various functional groups such as carboxylic acids, amines, amides, chlorides, nitriles, among others (Blotny, 2006; Giacomelli *et al.*, 2004). These reactions allow the engineering of novel derivative compounds which exhibit markedly different properties from their precursors. Hence, the isolated products can be ultimately employed in various areas such as in pharmaceutical sciences, in the textile industry, and in analytical chemistry. Following our interest in crystal engineering (Vilela *et al.*, 2009; Shi *et al.*, 2008; Paz & Klinowski, 2003, 2007; Paz *et al.*, 2002, 2005), we started using 2,4,6-trichloro-1,3,5-triazine as a molecular canvas for the preparation of novel multipodal organic ligands. A search in the literature and in the Cambridge Structural Database (CSD, Version of November 2008 with three updates; Allen, 2002) shows that the group of Bai (Wang, Xing *et al.*, 2007; Wang, Bai, Xing *et al.*, 2007; Wang, Bai, Li *et al.*, 2007) reported the only known examples of transition metal coordination polymers containing *N*,*N'*,*N''*-1,3,5-triazine-2,4,6-triyltrisglycine. We intend to further develop their concept by preparing mono-, di- and tri-substitued derivatives with several amino acid pendant groups. By using glycine methyl ester hydrochloride (Vilela *et al.*, 2009) we isolated the pure title compound (*i.e.*, the monosubstituted derivative, I).

At 150 K compound (I) contains two identical molecular units in the asymmetric unit (Fig. 1). The bond lengths and angles observed for the two molecules are statistically identical. The pendant methoxycarbonyl group exhibits considerable conformational flexibility due to the possibility of rotation around the —CH_2_— moiety. Indeed, while the rings and the —NH— moiety of the two crystallographically independent molecular units are almost co-planar, the pendant group is rotated by *ca* 180° (Fig. 2), with this feature arising with the objective to minimize steric repulsion in the crystal structure (see below).

The co-planarity of the —NH— bond with the ring of each molecular unit seems to be promoted by the existence of two strong (*d*_D···A_ being *ca* 3.02 Å) and highly directional [<(DHA) angles above 170° - see Table 1] N—H···N hydrogen bonding interactions that form a *R_2_^2^(8)* graph set motif (Bernstein *et al.*, 1995). This arrangement leads to the existence of supramolecular dimers (one for each molecular unit) in the crystal structure, with Fig. 3 depicting one of these. The close packing in the solid-state is based on the spatial interdigitation of the two dimers to effectively occupy the available space, hence the two conformations for the pendant groups which ultimately help promoting a more effective packing (Fig. 4).

### Experimental

Glycine methyl ester hydrochloride (193 mg, 2.169 mmol; Sigma-Adrich, 99%) and potassium carbonate (200 mg, 1.447 mmol; Sigma-Aldrich, >99.0%) were added at 273 K to a solution of 2,4,6-trichloro-1,3,5-triazine (100 mg, 0.542 mmol; Sigma-Aldrich, >98,0%) in dried toluene (*ca* 5 ml). The reaction mixture was kept under magnetic stirring and slowly heated to reflux under an anhydrous atmosphere. The reaction was controlled by TLC and stopped after 24 h. The reaction mixture was separated by flash column chromatography using as eluent a gradient of methanol in dichloromethane. The first isolated fraction was identified as (I) (7% yield). Single crystals were isolated from recrystallization of the crude product from a solution in dichloromethane: methanol (*ca* 1: 1). All employed solvents were of analytical grade and purchased from commercial sources.

^1^H NMR (300.13 MHz, CDCl_3_) δ: 3.83 (*s*, 3H, OCH_3_), 4.27 (*d*, 2H, *J* = 2.7 Hz, CH_2_), 6.35 (*br s*, 1H, NH). ^13^C NMR (75.47 MHz, CDCl_3_) δ: 42.8 (CH_2_), 52.8 (OCH_3_), 165.8 (CNH), 168.9 (CCl), 170.2 (CCl), 171.1 (CO_2_Me). MS (TOF MS ES+)*m/z*: 237.0 (*M*+H)^+^. Selected FT—IR data (ATR, in cm^-1^): ν(N—H) = 3264*m*; ν*_asym_*(—CH_3_) = 2961*m*; ν(C=O) = 1751*vs*; ν*_in-plane_*(ring) = 1549*s* and 1524*s* (doublet); δ(—CH_3_) = 1417*m*; ν(C_aromatic_—N) = 1322*m*; ν*_asym_*(C—O—C) = 1205*s*; ν*_sym_*(C—O—C) = 1134*s*; γ(ring) = 841*s*.

### Refinement

Hydrogen atoms bound to carbon were located at their idealized positions and were included in the model in the riding model approximation with C—H = 0.99 Å (for the —CH_2_— groups) or 0.98 Å (for the —CH_3_ moieties). The isotropic thermal displacement parameters for these atoms were fixed at 1.2 (methylene) or 1.5 (methyl) times *U*_eq_ of the carbon atom to which they are attached. The N—H atoms were located from difference Fourier maps and included in the structure with the N—H distance restrained to 0.95 (1) Å and with *U*_iso_ fixed at 1.5 times *U*_eq_ of the N atom.

The structure contains a large residual electron density of 1.78 e^.^Å^-3^ located at 1.36 Å of H4A. Attempts to include this peak as a disordered C atom did not lead to sensible structural refinements.

### Crystal data

**Table:**

C_6_H_6_Cl_2_N_4_O_2_	*Z* = 4
*M**_r_* = 237.05	*F*(000) = 480
Triclinic, *P*1	*D*_x_ = 1.654 Mg m^−^^3^
Hall symbol: -P 1	Mo *K*α radiation, λ = 0.71073 Å
*a* = 7.3543 (4) Å	Cell parameters from 6830 reflections
*b* = 9.7523 (5) Å	θ = 2.8–28.9°
*c* = 13.4133 (7) Å	µ = 0.66 mm^−^^1^
α = 97.714 (3)°	*T* = 150 K
β = 92.714 (3)°	Plate, colourless
γ = 90.225 (3)°	0.18 × 0.16 × 0.04 mm
*V* = 952.19 (9) Å^3^	

### Data collection

**Table:**

Bruker X8 Kappa CCD APEXII diffractometer	5043 independent reflections
Radiation source: fine-focus sealed tube	3753 reflections with *I* > 2σ(*I*)
graphite	*R*_int_ = 0.047
ω and φ scans	θ_max_ = 29.1°, θ_min_ = 3.7°
Absorption correction: multi-scan (*SADABS*; Sheldrick, 1997)	*h* = −10→10
*T*_min_ = 0.890, *T*_max_ = 0.974	*k* = −13→13
23605 measured reflections	*l* = −18→18

### Refinement

**Table:**

Refinement on *F*^2^	Primary atom site location: structure-invariant direct methods
Least-squares matrix: full	Secondary atom site location: difference Fourier map
*R*[*F*^2^ > 2σ(*F*^2^)] = 0.059	Hydrogen site location: mixed
*wR*(*F*^2^) = 0.165	H atoms treated by a mixture of independent and constrained refinement
*S* = 1.04	*w* = 1/[σ^2^(*F*_o_^2^) + (0.0886*P*)^2^ + 1.283*P*] where *P* = (*F*_o_^2^ + 2*F*_c_^2^)/3
5043 reflections	(Δ/σ)_max_ = 0.001
261 parameters	Δρ_max_ = 1.78 e Å^−^^3^
2 restraints	Δρ_min_ = −0.42 e Å^−^^3^

### Special details

**Table:**

Geometry. All e.s.d.'s (except the e.s.d. in the dihedral angle between two l.s. planes) are estimated using the full covariance matrix. The cell e.s.d.'s are taken into account individually in the estimation of e.s.d.'s in distances, angles and torsion angles; correlations between e.s.d.'s in cell parameters are only used when they are defined by crystal symmetry. An approximate (isotropic) treatment of cell e.s.d.'s is used for estimating e.s.d.'s involving l.s. planes.
Refinement. Refinement of *F*^2^ against ALL reflections. The weighted *R*-factor *wR* and goodness of fit *S* are based on *F*^2^, conventional *R*-factors *R* are based on *F*, with *F* set to zero for negative *F*^2^. The threshold expression of *F*^2^ > σ(*F*^2^) is used only for calculating *R*-factors(gt) *etc*. and is not relevant to the choice of reflections for refinement. *R*-factors based on *F*^2^ are statistically about twice as large as those based on *F*, and *R*- factors based on ALL data will be even larger.

### Fractional atomic coordinates and isotropic or equivalent isotropic displacement parameters (Å^2^)

**Table:**

	*x*	*y*	*z*	*U*_iso_*/*U*_eq_	
Cl1	0.03910 (11)	0.76811 (9)	0.64289 (6)	0.02802 (19)	
Cl2	0.05165 (10)	0.83098 (7)	1.03313 (5)	0.02279 (18)	
Cl3	0.55099 (12)	0.59177 (9)	0.63658 (6)	0.0312 (2)	
Cl4	0.54953 (10)	0.68082 (7)	1.02721 (6)	0.02374 (18)	
N1	0.0584 (3)	0.8055 (2)	0.83787 (18)	0.0195 (5)	
N2	0.3083 (3)	0.9206 (2)	0.93391 (18)	0.0172 (5)	
N3	0.3084 (3)	0.8875 (2)	0.75349 (18)	0.0177 (5)	
N4	0.5544 (3)	0.9858 (2)	0.85124 (18)	0.0182 (5)	
H4	0.610 (5)	1.014 (4)	0.9155 (14)	0.027*	
N5	0.5630 (3)	0.6316 (2)	0.83184 (19)	0.0214 (5)	
N6	0.8095 (3)	0.5542 (2)	0.93194 (17)	0.0160 (5)	
N7	0.8146 (3)	0.5166 (2)	0.75176 (18)	0.0178 (5)	
N8	1.0585 (3)	0.4599 (2)	0.85293 (18)	0.0174 (5)	
H8	1.109 (5)	0.456 (4)	0.9184 (13)	0.026*	
C1	0.1492 (4)	0.8273 (3)	0.7573 (2)	0.0179 (5)	
C2	0.1510 (4)	0.8563 (3)	0.9232 (2)	0.0173 (5)	
C3	0.3874 (4)	0.9307 (3)	0.8456 (2)	0.0163 (5)	
C4	0.6573 (4)	0.9941 (3)	0.7636 (2)	0.0194 (6)	
H4A	0.6299	0.9116	0.7136	0.023*	
H4B	0.7888	0.9934	0.7830	0.023*	
C5	0.6151 (4)	1.1235 (3)	0.7156 (2)	0.0175 (5)	
C6	0.6440 (5)	1.2261 (4)	0.5677 (3)	0.0321 (8)	
H6A	0.5217	1.2643	0.5759	0.048*	
H6B	0.6629	1.1990	0.4959	0.048*	
H6C	0.7349	1.2963	0.5957	0.048*	
C7	0.6559 (4)	0.5773 (3)	0.7536 (2)	0.0194 (6)	
C8	0.6527 (4)	0.6144 (3)	0.9185 (2)	0.0172 (5)	
C9	0.8913 (4)	0.5102 (3)	0.8450 (2)	0.0156 (5)	
C10	1.1643 (4)	0.4189 (3)	0.7663 (2)	0.0181 (5)	
H10A	1.2954	0.4260	0.7871	0.022*	
H10B	1.1405	0.4833	0.7162	0.022*	
C11	1.1208 (4)	0.2724 (3)	0.7175 (2)	0.0175 (5)	
C12	1.1656 (5)	0.1125 (3)	0.5745 (2)	0.0296 (7)	
H12A	1.2338	0.0502	0.6137	0.044*	
H12B	1.2142	0.1072	0.5073	0.044*	
H12C	1.0368	0.0849	0.5682	0.044*	
O1	0.6625 (3)	1.1059 (2)	0.62027 (16)	0.0241 (5)	
O2	0.5510 (3)	1.2274 (2)	0.75767 (17)	0.0289 (5)	
O3	1.1833 (3)	0.2526 (2)	0.62497 (15)	0.0222 (4)	
O4	1.0454 (3)	0.1858 (2)	0.75626 (17)	0.0298 (5)	

### Atomic displacement parameters (Å^2^)

**Table:**

	*U*^11^	*U*^22^	*U*^33^	*U*^12^	*U*^13^	*U*^23^
Cl1	0.0294 (4)	0.0362 (4)	0.0181 (4)	−0.0088 (3)	−0.0034 (3)	0.0043 (3)
Cl2	0.0263 (4)	0.0243 (3)	0.0186 (3)	−0.0070 (3)	0.0063 (3)	0.0042 (3)
Cl3	0.0374 (5)	0.0337 (4)	0.0217 (4)	0.0070 (3)	−0.0087 (3)	0.0045 (3)
Cl4	0.0263 (4)	0.0231 (3)	0.0221 (4)	0.0093 (3)	0.0070 (3)	0.0016 (3)
N1	0.0204 (12)	0.0204 (11)	0.0188 (12)	−0.0048 (9)	0.0021 (10)	0.0058 (9)
N2	0.0194 (12)	0.0155 (10)	0.0173 (11)	−0.0006 (9)	0.0032 (9)	0.0041 (9)
N3	0.0194 (12)	0.0180 (11)	0.0165 (11)	0.0009 (9)	0.0011 (9)	0.0045 (9)
N4	0.0176 (12)	0.0208 (11)	0.0163 (11)	−0.0014 (9)	0.0026 (9)	0.0017 (9)
N5	0.0217 (13)	0.0195 (11)	0.0223 (13)	0.0046 (10)	−0.0028 (10)	0.0012 (9)
N6	0.0179 (11)	0.0140 (10)	0.0161 (11)	0.0015 (8)	0.0017 (9)	0.0015 (8)
N7	0.0220 (12)	0.0167 (11)	0.0146 (11)	−0.0015 (9)	0.0013 (9)	0.0017 (8)
N8	0.0190 (12)	0.0186 (11)	0.0144 (11)	0.0020 (9)	0.0017 (9)	0.0010 (9)
C1	0.0212 (14)	0.0182 (12)	0.0143 (13)	0.0001 (10)	−0.0026 (10)	0.0030 (10)
C2	0.0198 (13)	0.0168 (12)	0.0161 (13)	−0.0009 (10)	0.0039 (10)	0.0040 (10)
C3	0.0190 (13)	0.0138 (11)	0.0170 (13)	0.0031 (10)	0.0021 (10)	0.0048 (10)
C4	0.0185 (13)	0.0215 (13)	0.0190 (14)	0.0020 (10)	0.0046 (11)	0.0040 (11)
C5	0.0160 (13)	0.0193 (12)	0.0170 (13)	−0.0012 (10)	0.0035 (10)	0.0007 (10)
C6	0.043 (2)	0.0308 (16)	0.0269 (17)	0.0052 (14)	0.0134 (15)	0.0146 (14)
C7	0.0263 (15)	0.0153 (12)	0.0169 (13)	−0.0008 (11)	−0.0031 (11)	0.0043 (10)
C8	0.0185 (13)	0.0137 (12)	0.0191 (13)	0.0016 (10)	0.0033 (10)	0.0002 (10)
C9	0.0185 (13)	0.0107 (11)	0.0171 (13)	−0.0024 (9)	0.0017 (10)	0.0003 (9)
C10	0.0176 (13)	0.0186 (12)	0.0177 (13)	−0.0016 (10)	0.0043 (10)	−0.0006 (10)
C11	0.0154 (13)	0.0209 (13)	0.0160 (13)	0.0006 (10)	0.0001 (10)	0.0015 (10)
C12	0.0411 (19)	0.0255 (15)	0.0194 (15)	0.0042 (13)	0.0031 (13)	−0.0075 (12)
O1	0.0330 (12)	0.0227 (10)	0.0178 (10)	0.0049 (9)	0.0089 (9)	0.0046 (8)
O2	0.0384 (13)	0.0257 (11)	0.0242 (12)	0.0106 (10)	0.0135 (10)	0.0047 (9)
O3	0.0316 (12)	0.0195 (10)	0.0152 (10)	0.0002 (8)	0.0057 (8)	−0.0006 (8)
O4	0.0389 (13)	0.0254 (11)	0.0252 (12)	−0.0109 (10)	0.0121 (10)	0.0002 (9)

### Geometric parameters (Å, °)

**Table:**

Cl1—C1	1.728 (3)	N8—C10	1.442 (4)
Cl2—C2	1.723 (3)	N8—H8	0.943 (10)
Cl3—C7	1.739 (3)	C4—C5	1.519 (4)
Cl4—C8	1.725 (3)	C4—H4A	0.9900
N1—C1	1.337 (4)	C4—H4B	0.9900
N1—C2	1.337 (4)	C5—O2	1.200 (4)
N2—C2	1.306 (4)	C5—O1	1.331 (3)
N2—C3	1.359 (4)	C6—O1	1.451 (4)
N3—C1	1.314 (4)	C6—H6A	0.9800
N3—C3	1.354 (4)	C6—H6B	0.9800
N4—C3	1.333 (4)	C6—H6C	0.9800
N4—C4	1.439 (4)	C10—C11	1.516 (4)
N4—H4	0.945 (10)	C10—H10A	0.9900
N5—C7	1.330 (4)	C10—H10B	0.9900
N5—C8	1.340 (4)	C11—O4	1.197 (4)
N6—C8	1.312 (4)	C11—O3	1.334 (3)
N6—C9	1.357 (4)	C12—O3	1.444 (4)
N7—C7	1.311 (4)	C12—H12A	0.9800
N7—C9	1.357 (4)	C12—H12B	0.9800
N8—C9	1.330 (4)	C12—H12C	0.9800
			
C1—N1—C2	111.0 (2)	O1—C6—H6B	109.5
C2—N2—C3	114.0 (2)	H6A—C6—H6B	109.5
C1—N3—C3	113.1 (2)	O1—C6—H6C	109.5
C3—N4—C4	122.6 (2)	H6A—C6—H6C	109.5
C3—N4—H4	119 (2)	H6B—C6—H6C	109.5
C4—N4—H4	119 (2)	N7—C7—N5	129.7 (3)
C7—N5—C8	110.5 (2)	N7—C7—Cl3	115.6 (2)
C8—N6—C9	113.7 (2)	N5—C7—Cl3	114.6 (2)
C7—N7—C9	113.1 (2)	N6—C8—N5	128.6 (3)
C9—N8—C10	122.3 (2)	N6—C8—Cl4	115.4 (2)
C9—N8—H8	117 (2)	N5—C8—Cl4	116.0 (2)
C10—N8—H8	120 (2)	N8—C9—N6	117.2 (2)
N3—C1—N1	129.1 (3)	N8—C9—N7	118.7 (2)
N3—C1—Cl1	116.2 (2)	N6—C9—N7	124.1 (3)
N1—C1—Cl1	114.7 (2)	N8—C10—C11	112.5 (2)
N2—C2—N1	128.4 (3)	N8—C10—H10A	109.1
N2—C2—Cl2	115.8 (2)	C11—C10—H10A	109.1
N1—C2—Cl2	115.8 (2)	N8—C10—H10B	109.1
N4—C3—N3	118.5 (3)	C11—C10—H10B	109.1
N4—C3—N2	117.2 (3)	H10A—C10—H10B	107.8
N3—C3—N2	124.3 (3)	O4—C11—O3	124.6 (3)
N4—C4—C5	112.4 (2)	O4—C11—C10	125.6 (3)
N4—C4—H4A	109.1	O3—C11—C10	109.8 (2)
C5—C4—H4A	109.1	O3—C12—H12A	109.5
N4—C4—H4B	109.1	O3—C12—H12B	109.5
C5—C4—H4B	109.1	H12A—C12—H12B	109.5
H4A—C4—H4B	107.9	O3—C12—H12C	109.5
O2—C5—O1	124.8 (3)	H12A—C12—H12C	109.5
O2—C5—C4	125.2 (3)	H12B—C12—H12C	109.5
O1—C5—C4	109.9 (2)	C5—O1—C6	115.8 (2)
O1—C6—H6A	109.5	C11—O3—C12	114.9 (2)
			
C3—N3—C1—N1	0.7 (4)	C8—N5—C7—N7	−1.6 (4)
C3—N3—C1—Cl1	−179.70 (19)	C8—N5—C7—Cl3	179.3 (2)
C2—N1—C1—N3	0.7 (4)	C9—N6—C8—N5	3.2 (4)
C2—N1—C1—Cl1	−178.9 (2)	C9—N6—C8—Cl4	−176.48 (19)
C3—N2—C2—N1	−2.7 (4)	C7—N5—C8—N6	−0.1 (4)
C3—N2—C2—Cl2	176.07 (19)	C7—N5—C8—Cl4	179.6 (2)
C1—N1—C2—N2	0.5 (4)	C10—N8—C9—N6	−175.6 (2)
C1—N1—C2—Cl2	−178.3 (2)	C10—N8—C9—N7	3.4 (4)
C4—N4—C3—N3	−3.4 (4)	C8—N6—C9—N8	173.8 (2)
C4—N4—C3—N2	175.9 (2)	C8—N6—C9—N7	−5.1 (4)
C1—N3—C3—N4	175.9 (2)	C7—N7—C9—N8	−175.2 (2)
C1—N3—C3—N2	−3.4 (4)	C7—N7—C9—N6	3.7 (4)
C2—N2—C3—N4	−175.1 (2)	C9—N8—C10—C11	−84.5 (3)
C2—N2—C3—N3	4.3 (4)	N8—C10—C11—O4	−18.0 (4)
C3—N4—C4—C5	86.3 (3)	N8—C10—C11—O3	163.6 (2)
N4—C4—C5—O2	21.8 (4)	O2—C5—O1—C6	4.0 (4)
N4—C4—C5—O1	−159.3 (2)	C4—C5—O1—C6	−174.9 (3)
C9—N7—C7—N5	−0.1 (4)	O4—C11—O3—C12	−4.2 (4)
C9—N7—C7—Cl3	178.98 (19)	C10—C11—O3—C12	174.2 (2)

### Hydrogen-bond geometry (Å, °)

**Table:**

*D*—H···*A*	*D*—H	H···*A*	*D*···*A*	*D*—H···*A*
N4—H4···N2^i^	0.95 (1)	2.09 (1)	3.028 (3)	171 (3)
N8—H8···N6^ii^	0.94 (1)	2.08 (1)	3.022 (3)	173 (3)

Symmetry codes: (i) −*x*+1, −*y*+2, −*z*+2; (ii) −*x*+2, −*y*+1, −*z*+2.
